# Editorial: Bone targeting nanoparticle drug delivery system in bone metabolism and bone-related tumor diseases

**DOI:** 10.3389/fphar.2022.1016631

**Published:** 2022-09-20

**Authors:** Xing Wang, Fenghua Meng, Zhiyong Lei, Daoyang Fan, Bo Lou

**Affiliations:** ^1^ Beijing National Laboratory for Molecular Sciences, Institute of Chemistry, Chinese Academy of Sciences, Beijing, China; ^2^ University of Chinese Academy of Sciences, Beijing, China; ^3^ Biomedical Polymers Laboratory, Soochow University, Suzhou, China; ^4^ Experimental Cardiology Laboratory/Central Diagnostic Laboratory, University Medical Center Utrecht, Utrecht, Netherlands; ^5^ The First Affiliated Hospital of Zhengzhou University, Zhengzhou University, Zhengzhou, China; ^6^ Department of Surgery, Yong Loo Lin School of Medicine, National University of Singapore, Singapore, Singapore

**Keywords:** functionalized bone targeting nanomaterials, bone-targeting molecules and mechanism, pharmacokinetics of therapeutic nanomaterials, nanomedicine toxicology for bone-related diseases, new nanotherapeutic strategy for bone-related diseases, nanomaterial-cell interaction for bone-related diseases

Bone is an essential mechanical support organ for organisms and a vital metabolic and immune-related organ. The bone formation process involves mesenchymal stem cells, osteoblasts, osteoclasts, osteoid, nuclear factor receptors, osteoprotegerin, and macrophage colony-stimulating factor. The highly specified dynamic tissue is constantly being metabolized and remodeled throughout life to maintain a healthy skeletal structure for these functions. Bone metabolism involves multiple basic bone cells that act as key regulators, including osteocytes, osteoblasts, and osteoclasts, which either on their own or in interaction keep the balance between bone catabolism and anabolism. Changes in the number or function of any part would inevitably destroy the emotional balance of bone metabolism and cause osteoporosis, Paget’s disease, metastatic bone tumors, and other diseases in severe cases. The concept of “bone targeting” developed by Pierce in 1986 aimed to increase the drug concentration of target cells or target organs and reduce the systemic adverse drug reactions by reducing drug delivery and lowering the drug concentration of non-target organs. Targeted delivery by either the systemic or local targeting of therapeutics to the bone is an attractive treatment for various bone metabolism diseases such as osteoporosis, osteoarthritis, osteosarcoma, and osteomyelitis, etc. Researchers have proposed many strategies for the targeted treatment of bone metabolism diseases, including bone-targeted nanomaterials to perform chemotherapy, photothermal therapy, gene therapy, and combination therapy.

This Research Topic brings together four articles written by 29 authors, containing two reviews and two original research articles. Review articles mainly presented two up-to-date aspects of systemic or local bone-targeting approaches, nano-therapeutic strategies, and nano-system applications in bone-related diseases.

Chen et al. summarize current advances in systemic or local bone-targeting approaches and nano-system applications in bone diseases, which provided new insights into nanocarrier-delivered drugs for the targeted treatment of bone diseases of clinical applications. Guo et al. summarize the recent advancements in new nanotherapeutic strategies for osteoarthritis including nanotechnologies for small molecule drug delivery, nanoparticles for biomacromolecule therapy, cell-based nanotherapeutic strategies, and other functional nanomaterials, and provide many suggestions for improving the treatment of osteoarthritis by means of the nanomaterials and intelligent nanomedicines.

In the original research paper contributions, researchers focused on specific biomaterials and strategies for bone treatments. Izzah Ibrahim et al. combined the tocotrienol with a nanocarrier of poly lactic-co-glycolic acid (PLGA) to inject into the bones of ovariectomized rats. The results showed great improvement in bone strength by the significantly higher stress, strain, stiffness, and Young’s modulus parameters, which achieved the targeted and controlled delivery of tocotrienol into the bone microenvironment for osteoporosis therapy. In addition, Zhang et al. incorporated an effectively chondro-inductive non-protein bioactive drug molecule into a naturally-derived composite hydrogel to fabricate appropriate microenvironments of BMSCs for cartilage regeneration. *In vitro* results showed that this composite hydrogel possessed excellent biocompatibility for facilitating cell growth, adhesion, proliferation, and differentiation. Although it is used for chondrogenic differentiation and cartilage repair, the long-term sustainable drug release from the hydrogel scaffolds *in situ* provided an appropriate and universal strategy for tissue regeneration by delivering the stable bioactive promoter locally, which exhibits great potential for clinical tissue regeneration.

The grand aim of this Research Topic is to bring together cutting-edge research on bone-targeted nanoparticle drug delivery systems for the treatment of bone metabolism and bone-related tumor diseases through nano-level pharmacology, which present unique characteristics enabling access to a wealth of advanced biomaterials for application in the tissue engineering and regenerative medicine. We sincerely hope that you will enjoy reading all the papers in this special edition.

